# Gene Flow among Populations of Two Rare Co-Occurring Fern Species Differing in Ploidy Level

**DOI:** 10.1371/journal.pone.0045855

**Published:** 2012-09-20

**Authors:** Anna Bucharová, Zuzana Münzbergová

**Affiliations:** 1 Department of Botany, Faculty of Science, Charles University, Prague, Czech Republic; 2 Institute of Botany, Academy of Science, Průhonice, Czech Republic; 3 Department of Plant Ecology, Institute of Evolution and Ecology, University Tübingen, Tübingen, Germany; University of Oxford, United Kingdom

## Abstract

Differences in ploidy levels among different fern species have a vast influence on their mating system, their colonization ability and on the gene flow among populations. Differences in the colonization abilities of species with different ploidy levels are well known: tetraploids, in contrast to diploids, are able to undergo intra-gametophytic selfing. Because fertilization is a post-dispersal process in ferns, selfing results in better colonization abilities in tetraploids because of single spore colonization. Considerably less is known about the gene flow among populations of different ploidy levels. The present study examines two rare fern species that differ in ploidy. While it has already been confirmed that tetraploid species are better at colonizing, the present study focuses on the gene flow among existing populations. We analyzed the genetic structure of a set of populations in a 10×10 km study region using isoenzymes. Genetic variation in tetraploid species is distributed mainly among populations; the genetic distance between populations is correlated with the geographical distance, and larger populations host more genetic diversity than smaller populations. In the diploid species, most variability is partitioned within populations; the genetic distance is not related to geographic distance, and the genetic diversity of populations is not related to the population size. This suggests that in tetraploid species, which undergo selfing, gene flow is limited. In contrast, in the diploid species, which experience outcrossing, gene flow is extensive and the whole system behaves as one large population. Our results suggest that in ferns, the ability to colonize new habitats and the gene flow among existing populations are affected by the mating system.

## Introduction

Gene flow is the successful movement of genes among populations by mating or by the migration of diaspores, and it is one of the key factors determining the spatial genetic structure of populations [1], [2]. Gene flow is usually considered beneficial for population survival, preventing inbreeding depression and the loss of genetic variation in small populations due to genetic drift [3]. In some cases, gene flow can also be detrimental for small populations because it prevents differentiation through the local adaptations of populations in different extreme conditions and reduces individual fitness through outbreeding depression [4].

The intensity of gene flow is, to a vast degree, influenced by the mating system of the species [5], [6]. The effects of the mating system on the intensity of gene flow has been extensively studied in seed plants (meta-analysis in [6]), and it was shown that gene flow among populations increases with an increasing level of outcrossing [6]. Compared to seed plants, the breeding system in ferns is more complex due to a specific life cycle involving independent haploid and diploid phases. Three types of fertilization occur in ferns [7]: (i) intra-gametophythic selfing (the fusion of sperm and egg from the same gametophyte resulting in a complete homozygote); (ii) inter-gametophytic selfing (the fusion of sperm and egg from different gametophytes derived from the same parental sporophyte, which is equivalent to selfing in seed plants); and (iii) outcrossing (the fusion of sperm and egg from gametophytes derived from spores of different sporophytes).

The fact that fertilization in ferns occurs on the gametophyte, which originates from spores, is of crucial importance for dispersal. In seed plants, fertilization takes place prior to dispersal, before the seed is formed. Theoretically, one seed is enough to colonize a new habitat. In ferns, fertilization is a post dispersal process on the haploid gametophyte. For ferns unable to use intra-gametophytic selfing, this means that the two spores must fall in close proximity and under favorable conditions on a new habitat. Fertilization can only occur after a gametophyte with archegonia/anteridia develops from both. Colonization of a new habitat is problematic in this case [8]. Spores transported long distances are unlikely to establish close enough to allow for inter-gametophytic selfing or crossing, despite the huge amount of spores produced by ferns. On the other hand, if the species is able to undergo intra-gametophytic selfing, one spore, which develops in a hermaphroditic gametophyte, is enough to establish a new, totally homozygous population. Many fern species that rely on intra-gametophytic selfing as the main breeding system have been shown to be great colonists on the continental scale [9]–[11].

The differences in colonization abilities naturally result in very different patterns of genetic variation. Thus, the structure of genetic diversity was used as a source of information about the mating system of ferns in many studies [12]–[16]. The type of mating system in ferns is often connected to the ploidy level of the species. The comparison of the distribution of genetic variation in populations of tetraploid species with diploid ancestors suggests that diploid species primarily undergo outcrossing and tetraploids primarily undergo selfing. Diploid parental species have a rather limited area with high genetic variability, while descendant tetraploids are widely distributed, but genetically uniform due to single spore colonization and inbreeding [15], [16].

Studies on the distribution of genetic diversity within and among populations are limited because it is difficult to distinguish between the gene flow among already established populations and historical processes during the colonization of new habitats ([17] but see [12]). Despite this difficulty, distinguishing between these two processes is crucial, and many studies have not paid proper attention to it [18]–[20]. During colonization of empty habitats, selfing species have a higher probability of establishing themselves in a vacant area and selfing, which strongly increases the probability of colonization. In contrast, outcrossing is more advantageous for gene flow between already established populations because it facilitates the incorporation of new genetic information into the gene pool of the given population, thus enhancing the gene flow among populations. To separate these two types of processes, information about the genetic structure of the population is not sufficient (but see [17]). Additional information about the colonization rates of the system are necessary because the current patterns of genetic diversity combine both processes that can occur during the different phases of population development.

In the present study, we analyzed the genetic structure of the populations to investigate gene flow among the populations of two rare fern species, *Asplenium adulterinum* and *A. cuneifolium*, which differ in both ploidy level and their breeding system. In our model system, the two species occupy very similar habitats – serpentine rocks, which are scattered in an area of 10×10 km. From our previous study, we have additional information about the colonization rates of the species in the system [21] and on the relative speed of the metapopulation dynamics of the species [22].

Our study addressed the following questions: 1) What is the distribution of genetic diversity within and among the populations of *Asplenium adulterinum* and *A. cuneifolium*?, 2) What is the intensity of gene flow among the populations? and 3) Do the patterns differ between species, and do they correspond to the expected difference in mating systems?

Furthermore, we compare the results from the current study about the genetic structure of the populations of the two species with information about the colonization rates in the system [21]. We discuss processes that might play a role during colonization and the subsequent gene flow among populations.

## Methods

### Study Species

The study involves two fern species, a tetraploid, *Asplenium adulterinum* Milde. and a diploid, *Asplenium cuneifolium* Viv. (Aspleniaceae), both of which are restricted to the serpentine substrate in Europe. Distribution of both species is highly scattered, following serpentine rocks in Europe from the Mediterranean to Norway and from Greece to Spain (*A. cuneifolium*, *A. adulterinum* is only found from Austria to France) [23]. In the study area, the Czech Republic, both species occur mainly in western Bohemia (Slavkovský les), with several localities in north-eastern Bohemia. There are only a few small populations found in the rest of the country. *A. cuneifolium* is generally more widespread than *A. adulterinum*.

Both species are rare and of interest to nature conservation groups throughout Europe [23]. Additionally, *A. adulterinum* is classified as a species of interest to the European ecological network Natura 2000 [24]. The species differ in ploidy level, *A. adulterinum* is allotetraploid (parental species *A. viride* L. and *A. trichomanes* Huds. subsp. *Trichomanes*), [25], wheras *A. cuneifolium* is diploid [26].

### Study Site

This study was carried out in the region of Slavkovský les in western Bohemia, Czech Republic. In this region of ca. 10 × 10 km, 98 serpentine rocks are scattered across the landscape [21]. The system is rather isolated from other populations of both species; the next closest population is 50 km away. Both *A. adulterinum* and *A. cuneifolium* are quite common in the area. *A. adulterinum* is more common and occupies rocks in both unforested habitat and the forest (dominated by *Pinus sylvestris* and *Picea abies*). In total, there are 66 populations located throughout the area, ranging from a few individuals to nearly 2000 individuals [27]. *A cuneifolium* prefers rocks under the forest canopy. The unforested rocks are rarely inhabited and the populations are very small. In total, there are 48 populations of *A. cuneifolium* in the study region, mostly in the central area, with several more distant localities, and the populations range from several individuals up to several hundred individuals [27].

For both species, unoccupied suitable habitats exist in the area; *A. adulterinum* occupies 81% of the suitable habitats and *A. cuneifolium* occupies 73%, indicating metapopulation dynamics in the study system [21].

### Sample Collection

Samples for isoenzyme analysis were collected from 14 localities in *A. adulterinum* and 12 localities in *A. cuneifolium*. Sampling design followed the distribution of the species in the study area (*A. adulterinum* has more populations). If available, 20 plants per population were sampled for each species. In total, we sampled 268 individuals of *A. adulterinum* and 227 individuals of *A. cuneifolium*.

Samples were evenly distributed over each locality to represent the entire range of variability within the population (under the assumption that geographically more distant plants are less closely related). We sampled 1–2 young leaves without spores per plant, being careful to not seriously damage the plant. The position of each plant was recorded using GPS (Global Position System) or marked on a map in the field, followed by digitalization of the map.

### Isoenzyme Analysis

Samples collected in the field were kept on ice for 24–48 hours until the isoenzymes were extracted in the laboratory. Electrophoresis was performed on the crude protein extracts of the leaf material. All enzymes were resolved on polyacrylamide gels using an 8.16% separating gel and a 4% stacking gel.

Nine enzymatic systems were studied, 7 of which provided an interpretable pattern and were variable at least for one of the study species: LAP, DIA, 6-PGDH, SHDH, PGM, ADH and AAT. For a detailed methodology of the isoenzyme extraction, electrophoresis and staining procedures, see Appendix S1.

### Band Interpretation

Bands were interpreted in two ways. First, only the presence or the absence of alleles was recorded, and the data were further treated as a dominant marker (for similar approach see, e.g., [28]). The dominant data approach was chosen because *A. adulterinum* is an allotetraploid, and it was rarely possible to assess the exact ratio of alleles from the intensity of the bands. Moreover, allotetraploids often have fixed pairs of alleles that always segregate together [11]. Thus, heritability may be disomic rather than tetrasomic, and it is often impossible to reliably distinguish which alleles segregate together as one de facto allele. As a result, recording only the presence or the absence of the alleles was the only appropriate way to treat the obtained pattern in all of the enzymatic systems. In *A. cuneifolium*, the same approach of treating the data as a dominant marker was used to compare the species.

We also evaluated the data as a co-dominant marker. This was possible for all enzymatic systems in *A. cuneifolium*, but only in one system in *A. adulterinum* – where we were able to distinguish how the alleles segregate in fixed pairs (see [11]). Because the data are very limited for *A. adulterinum*, it must be interpreted with caution. Despite this, the data brought interesting insight to the comparison of the mating systems of the two species.

### Statistical Analysis

#### Dominant marker

Binary (presence/absence) data were prepared in the program FAMD [29] and then imported into the program Arlequin [30], where most of the analyses were performed. A mantel test was performed using PopTools [31].

The mean gene diversity [32] was calculated for each population and averaged over all populations within a species. In addition, the number of haplotypes (band patterns) for each species and population was calculated. Distribution of genetic variability among and within populations was investigated using an AMOVA [33] and tested with a permutation test (1000 permutations). A Mantel test [34] was used to test for the relationship between pairwise F_st_ values between populations and pairwise geographic distance (in meters) between centroids of localities hosting the populations (obtained in study [21]). The relationship between the total size of the population and its genetic diversity was examined using a simple linear regression in the program R [35].

#### Co-dominant marker

We calculated the mean expected and observed heterozygosity and inbreeding coefficient [32] over all populations for both species using PopGene [36].

## Results

### Band Pattern

The 7 enzymatic systems provided a total of 9 interpretable loci: AAT, ADH-1, ADH-2, DIA, LAP-1, LAP-2, 6-PGHD, PGM and SHDH. In *A. adulterinum*, 4 loci were variable: LAP-1, DIA, 6-PGDH, SHDH. In *A. cuneifolium*, 7 loci were variable: AAT, ADH-1, ADH-2, LAP-1, LAP-2, PGM and SHDH. Because *A. cuneifolium* and *A. adulterinum* are not closely related species, the loci do not always correspond. However, for the purpose of these analyses, it is important to have the same number of loci for both species. Each of the 9 loci had 2 alleles, resulting in data matrix of 18 (presence/absence of an allele) × the total number of samples.

In tetraploid *A. adulterinum*, only one locus (LAP-1) could be reliably evaluated as allelic data. This locus either showed fixed heterozygosity (balanced pattern AABB) or fixed homozygosity (pattern AAAA), indicating diploid inheritance (due to a disomic heritability in allotetrapoids). Rarely, a clear pattern of AAAB was observed (in 2.6% of examined plants, see Appendix S2). This unbalanced pattern was interpreted as a heterozygote of fixed allele pairs AA and AB (see [11]). In diploid *A. cuneifolium*, all polymorphic enzymatic systems were evaluated as diploid allelic data.

### Dominant Marker

The mean gene diversity across the populations was 0.47 (ranging from 0.029 to 0.8) in tetraploid *A. adulterinum* and 0.94 (ranging from 0.93 to 0.99) in diploid *A. cuneifolium*. In *A. adulterinum*, only 14 haplotypes were present in the entire dataset, with separate populations containing 2–8 haplotypes. In *A. cuneifolium*, 96 haplotypes were present in the entire dataset, with separate populations containing 4–19 haplotypes.

**Figure 1:**
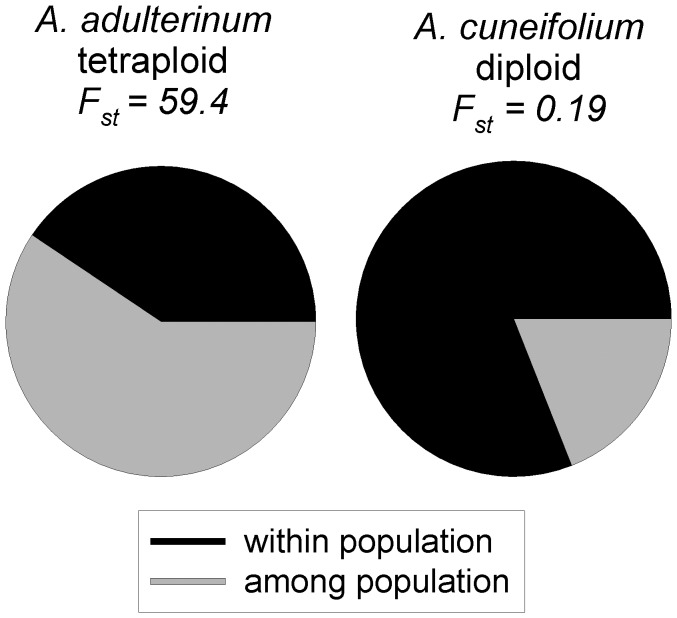
Partitioning of genetic variation within and among populations in the two species. From the results of AMOVA, the partitioning was highly significant in both species.

In *A. adulterinum*, 40.6% of genetic variation was within populations and 59.4% was among populations (F_st_ = 0.594, p<0.0001). It contrast, in *A. cuneifolium*, 81.0% of genetic variation was within populations and only 19.0% was among populations (F_st_ = 0.190, p<0.0001), Fig 1.

**Figure 2:**
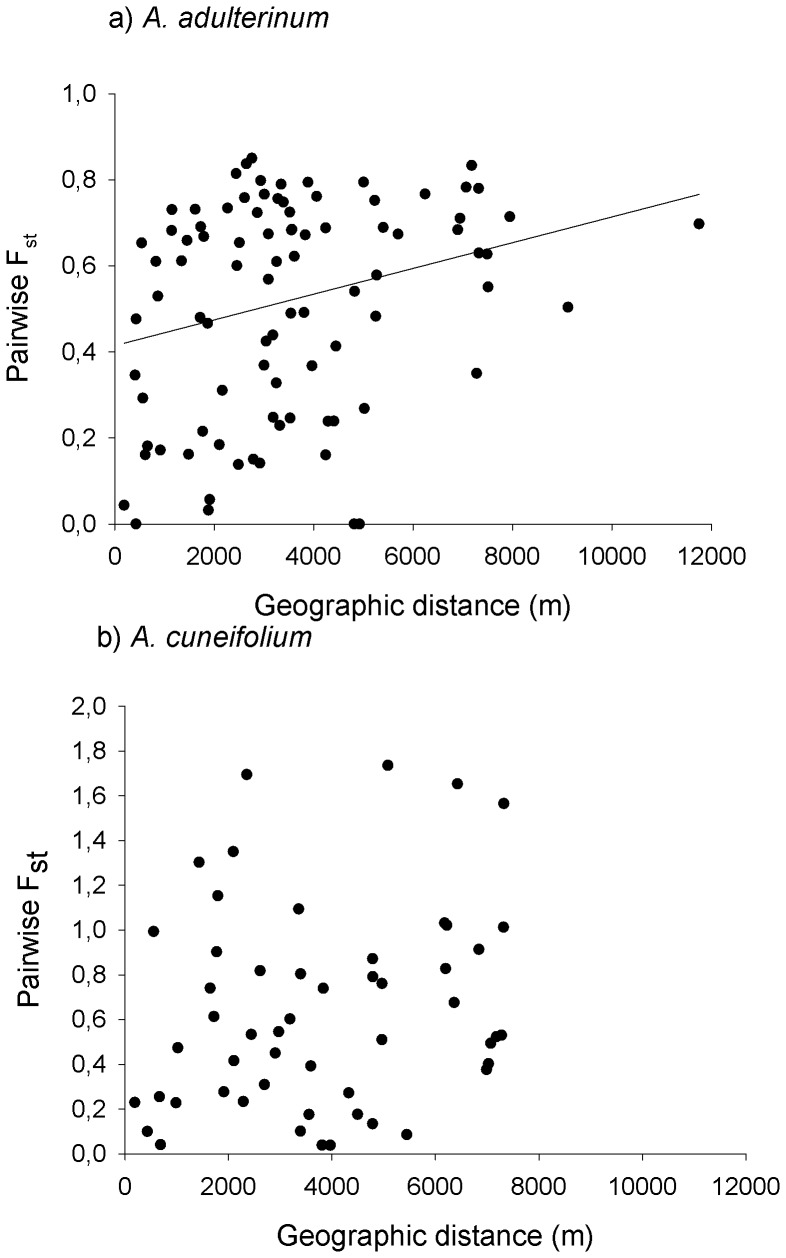
Relationship between geographic distance and pairwise F_st_ between populations. The relationship is significant in tetraploid *A. adulterinum* (Mantel r = 0.35, p = 0.001, A), but not in diploid *A. cuneifolium* (p = 0. 317, B). The relationship in *A. adulterinum* is significant also when the most remote population is removed from the analysis (Mantel r = 0.29, p = 0.035, B).

**Figure 3:**
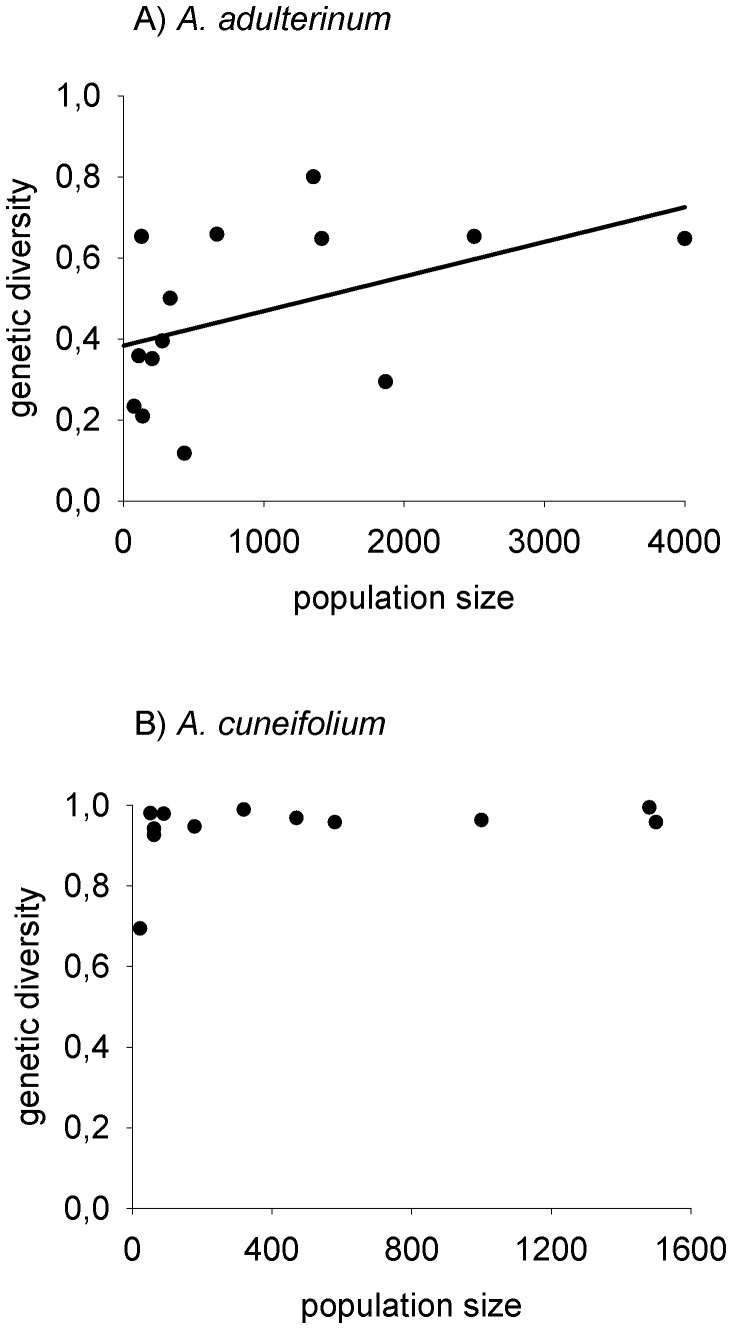
Relationship between population size and genetic diversity (Nei 1987) of individual populations. The relationship is marginally significant in tetraploid *A. adulterinum* (R^2^ = 0.152, p = 0.093, linear regression, A), but not in diploid *A. cuneifolium* (p = 0.290, B).

The correlation between geographic and genetic distance between populations was highly significant (Mantel r = 0.335, p = 0.001) in *A. adulterinum*. In contrast, in *A. cuneifolium*, the relationship was not significant (Mantel r = 0.093, p = 0.317), Fig 2. Larger populations of *A. adulterinum* host more genetic diversity. This relationship was, however, only marginally significant (R^2^ = 0.152, p = 0.093). In *A. cuneifolium*, no relationship between the size of the population and the genetic diversity was observed (p = 0.290), Fig 3.

### Co-dominant Marker

According to the one enzymatic system (LAP-1) that made allowance for reliable co-dominant scoring, there was very high deficiency of heterozygotes in *A. adulterinum*. As expected, the observed heterozygosity in *A. cuneifolium* was much more balanced in all enzymatic systems (Table 1).

**Table 1:** Expected (H_e_) and observed (H_o_) heterozygosity and inbreeding coefficient (F_is_) per locus.

	locus	H_e_	H_o_	F_is_
*A. adulterinum*	LAP - I	0.490	0.026	0.862
*A. cuneifolium*	AAT	0.419	0.489	0.041
	SHDH	0.145	0.179	0.076
	LAP - I	0.234	0.495	0.368
	LAP - II	0.031	0.039	0.144
	ADH - I	0.189	0.294	0.237
	ADH - II	0.212	0.452	0.375
	PGM	0.080	0.084	−0.129
	Mean over 7 loci	0.290	0.188	0.209

## Discussion

The present study revealed considerable differences in the genetic structure of two populations of rare fern species differing in ploidy level. Populations of the allotetraploid, *Asplenium adulterinum*, have a high genetic differentiation, and the difference increases with geographical distance. However, the individuals are rather uniform within populations. In contrast, populations of the diploid, *A. cuneifolium*, are very similar to each other, but individuals within populations are genetically variable. Based on these results, we can deduce the probable mating system of the species and the intensity of gene flow between populations. Together with the results from our previous study [21], we can discuss the probable mechanisms of colonization and gene flow among the populations of the two fern species.

### Mating System

Our original assumption about the differences in the mating system between *A. adulterinum* (4n) and *A. cuneifolium* (2n) was based on their ploidy level and on the common expectation that diploids undergo selfing and polyploids undergo outcrossing [37]. However, recent studies do not confirm this strict division (e.g., [12]). Thus, it was necessary to obtain independent data to confirm or reject this expectation.

The mating system of a species is commonly estimated from the genetic structure of its populations, where the genetic variation is partitioned mainly within populations of outcrossing species and among populations of selfing species [16], [38]–[39]. The results of our study corroborate those of previous studies – *Asplenium adulterinum*, which is expected to undergo selfing, has 59.4% of the genetic variability partitioned among its populations, while *A. cuneifolium*, which is an expected outcrossing species, has 19% of the genetic variability partitioned among populations (based on the dominant marker).

Additionally, the enzymatic system, in which interpretation based on alelles was possible, shows a striking lack of heterozygotes – we found only 2.6% of heterozygotes in *Asplenium adulterinum*. This suggests a high level of inbreeding. In contrast, the observed heterozygosity in *A. cuneifolium* was rather balanced, as expected. Therefore, the genetic population structure suggests that selfing is the prevailing mating system for *A. adulterinum*, while outcrossing is the prevailing mating system for *A. cuneifolium*.

Our data further showed that *A. adulterinum* is capable of outcrossing because we found heterozygotes, which are clearly a direct output from crossing two different genotypes. However, the frequency of individuals resulting from outcrossing is quite low. This finding corresponds to the conclusions of previous studies that gametophytic selfing is the main mating system in polyploid ferns [16], [38].

Unfortunately, our data cannot provide information regarding whether *A. cuneifolium*, the species with prevailing outcrossing, is capable of intragametophytic selfing, and thus, single spore colonization. However, single spore colonization has been confirmed in other diploid species, such as *Asplenium scolopendrium* [12]. This study [12] further suggested that the offspring originating from gametophytic selfing are competitively excluded (due to smaller vitality) by offspring that originated from outcrossing. A similar scenario may be expected in both of our study species: the genetic structure of the sporophyte population may not fully reflect the frequency of the type of gametophytic mating (selfing/outcrossing). Because both inbreeding and outbreeding depression was clearly documented in ferns [40], young sporophytes originating from selfing in predominantly outcrossing diploids (or from outcrossing in predominantly selfing polyploids) might be excluded from the population via competition.

### Gene Flow among Populations

Our study revealed that the genetic structure of the populations of the two species strongly differ on a regional spatial scale (ca 10 km). Populations of *A. adulterinum* show strong genetic differentiation similar to other predominantly selfing fern species, e.g., [10], [41]; the genetic distance between populations increases with the geographical distance, and larger populations contain more genetic variability. The gene flow among populations of this selfing species is rather limited. In contrast, in *A. cuneifolium*, there is no relationship between genetic and geographic distance, and most of the genetic diversity can be found within populations. This result suggests that the whole system of this species functions as one large population with frequent dispersal throughout the area and a high level of gene flow, as is often found in various outcrossing fern species (e.g., [42]–[44],but see also [45]).

### Probable Processes Forming Genetic Structure

When we combine the results of the present study about the genetic structure of the populations with information on the colonization ability of the species [21], we can hypothesize which processes form the genetic structure of the two fern species during colonization and the subsequent gene flow.

*A. adulterinum* is predominantly a good colonist of empty patches [21], but subsequent gene flow between the populations is rather limited. The genetic diversity of populations of this species is likely affected by the founder effect. The patch is occupied by the few genotypes that arrive, and they do not mix (or do to a very limited degree) with other genotypes. If outcrossing occurs, its product may be excluded due to outbreeding depression [46]. As a result, several independent genotypes exist on patches and reproduce mostly via selfing, leading to highly differentiated populations.

In comparison with *A. adulterinum*, *A. cuneifolium* has a more difficult time colonizing empty patches. However, outcrossing facilitates effective gene flow between already established populations because the new genotype must be crossed with the local gametophytes to be incorporated into the population’s gene pool (as suggested by Wubs [12]). Moreover, if inbreeding occurs in a predominantly outbreeding species, its product may have a disadvantage due to inbreeding depression [40] and may be excluded from the population. The resulting effective gene flow thus diminishes any spatial structure among populations.

## Supporting Information

Appendix S1 **Detailed methods of isoenzyme analysis.**
(DOC)

Appendix S2 **Unbalanced isoenzyme banding pattern AAAB in locus LAP-1 in tetraploid** ***A. adulterinum*** **.**
(DOC)
