# Supplementary material for: Gene Flow among Populations of Two Rare Co-Occurring Fern Species Differing in Ploidy Level
Source: PLoS One. 2012 Sep 20;7(9):e45855. doi: 10.1371/journal.pone.0045855 (PMC3447768; doi:10.1371/journal.pone.0045855)
Supplement: Appendix S1 — Detailed methods of isoenzyme analysis. (DOC) [file pone.0045855.s001.doc]

**Detailed protocol used for the isozyme analysis**

Electrophoresis was performed on crude protein extracts of leaf material. Approximately 60 mg of fresh leaf tissue was ground with Dowex-Cl (1-X8) and homogenized on ice in 0.6 ml Tris–HCl extraction buffer (0.1 M Tris–HCl pH 8.0, 78 mM 2-mercaptoethanol, 26 mM sodium metabisulfite, 11 mM ascorbic acid, 4% polyvinylpyrrolidone). The extracts were centrifuged for 10 min at 15,000 rpm and clear supernatants were stored at −75 °C for up to 6 months until electrophoresis. Isozymes were separated on native-PAGE; 15 μl of each sample were employed for electrophoresis in a Hoefer vertical electrophoresis unit. All enzymes were resolved on polyacrylamide gels using 8.16% separating gel and 4% stacking gel. The separating gel was made using a buffer of 1.82 M Tris–HCl, pH 8.9, and the stacking gel using a buffer of 0.069 M Tris–HCl, pH 6.9. The electrode buffer consisted of 0.02 M tris and 0.24 M glycine, pH 8.3.

Nine enzyme systems were investigated in the first step (6-PGDH, AAT, ADH, DIA, IDH, LAP, MDH, PGM, SHDH); variation was found in 4 of them in *A. adulterinum* (LAP, DIA, 6-PGDH, SHDH) and 5 of them in *A. cuneifolium* (AAT, SHDH, LAP 2 loci, ADH 2 loci, PGM), resulting in 4 and 7 variable loci, respectively.

The staining procedures followed Vallejos (1983) to visualize 6-PGDH, ADH, PGM, SHDH and DIA, with the following modifications: 6-PGDH (30 ml 0.1 M Tris–HCl pH 8.4, 10 mg 6-phosphogluconate, 5 mg NADP, 5 mg MTT, 2 mg PMS, 30 mg MgCl_2_), ADH (40 ml 0.1 M Tris-HCl pH 7.5, 30 mg NAD, 20 mg MTT, 2 mg PMS, 20 ml ethanol), PGM (50 ml 0.05 M Tris-HCl pH 8.5, 100 mg glucose-1-phosphate, 10 mg NADP, 10 mg MTT, 2 mg PMS, 25 mg MgCl_2_, 80 units glucose-6-phosphate dehydrogenase), SHDH (30 ml 0.1 M Tris-HCl pH 8.4, 30 mg shikimic acid, 5 mg NADP, 6 mg MTT, 2 mg PMS) and DIA (100 ml 0.1 M Tris-HCl pH 8.0, 4 mg 2,6-dichlorophenol-indophenol, 26 mg NADH, 10 mg MTT). Enzyme system AAT was stained using the following method: two staining solutions were prepared, A (20 ml 0.1 M Tris–HCl pH 8.4, 240 mg aspartic acid, 40 mg α-ketoglutaric acid) and B (20 ml 0.1 M Tris–HCl pH 8.4, 25 mg Fast Blue BB Salt, 50 mg Fast Violet B, 25 mg pyridoxal-5-phosphate). Solution A was prepared at least 15 min before the application. The gel was rinsed in water and then in buffer (Tris–HCl pH 7). Solutions A and B were mixed and poured on the gel. The gel was incubated in the dark at 35 °C until bands appeared. Then the gel was rinsed in distilled water and fixed (1:1:3:5, glycerine:acetic acid:H_2_O:methanol). Visualization of LAP was done using buffer 0.2 M Tris-maleat pH 6. The gel was rinsed with the buffer and then incubated for 10 min in a solution of 30 ml buffer, 50 mg L-leucyl-β-naphthylamide HCl (in 50% acetone) and 60 mg MgCl_2_. Then 25 mg Fast Black K Salt in 30 ml of the buffer was added. The gel was incubated in dark, until bands appeared.
