# Supplementary figures and images for: Gene Flow among Populations of Two Rare Co-Occurring Fern Species Differing in Ploidy Level
Source: PLoS One. 2012 Sep 20;7(9):e45855. doi: 10.1371/journal.pone.0045855 (PMC3447768; doi:10.1371/journal.pone.0045855)

Figure S2: Unbalanced isozyme banding pattern AAAB in locus LAP-1 in tetraploid *A. adulterinum*.


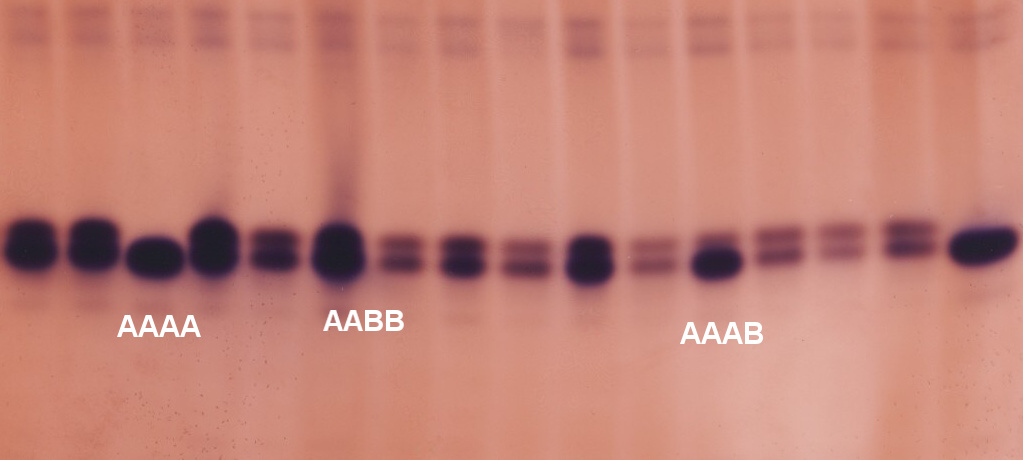

Supplement: Appendix S2 — Unbalanced isoenzyme banding pattern AAAB in locus LAP-1 in tetraploid A. adulterinum . (DOC) [file pone.0045855.s002.doc]
